# Dissecting Hidden Liraglutide Oligomerization Pathways via Direct Mass Technology, Electron-Capture Dissociation, and Molecular Dynamics

**DOI:** 10.1021/acs.analchem.5c01851

**Published:** 2025-06-16

**Authors:** Syuan-Ting Kuo, Zhenyu Xi, Xiao Cong, Xin Yan, David H. Russell

**Affiliations:** † Department of Chemistry, 14736 Texas A&M University, College Station, Texas 77843, United States; ‡ 6893 Boehringer Ingelheim, Ridgefield, Connecticut 06877, United States

## Abstract

Peptide therapeutics have revolutionized drug design strategies, yet the inherent structural flexibility and conjugated moieties of drug molecules present challenges in discovery, rational design, and manufacturing. Liraglutide, a GLP-1 receptor agonist conjugated with palmitic acid at its lysine residue, exemplifies these challenges by forming oligomers, which may compromise efficacy through the progressive formation of aggregates. Here, we incorporate native mass spectrometry platforms, including electron-capture dissociation (ECD), direct mass technology (DMT), and molecular dynamics (MD), to capture the early oligomerization process of liraglutide. Our findings reveal a restricted C-terminal region upon oligomer formation, as indicated by the reduced release of *z*-ions in ECD analysis. Additionally, we identified the formation of higher-order oligomers (*n* = 25–62) by DMT, primarily stabilized by hydrophilic interactions involving preformed stable oligomers (*n* = 12–18). Together, these integrative mass spectrometry results delineate a dual-pathway oligomerization process for liraglutide, demonstrating the power of mass spectrometry to uncover hidden pathways of self-association. This approach underscores the potential of mass spectrometry as a key tool in the rational design and optimization of peptide-based therapeutics.

## Graphical abstract

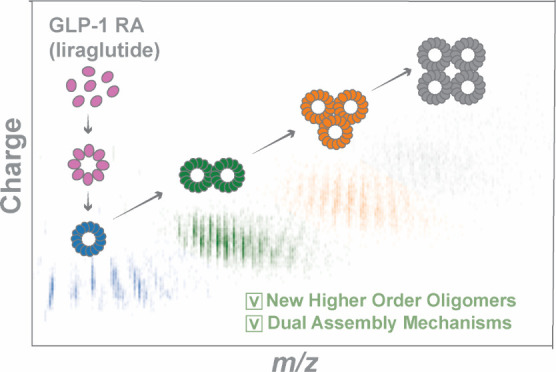

## Introduction

Liraglutide, a glucagon-like peptide-1 receptor agonist (GLP-1 RA) modified through the conjugation of a C16 fatty acid to Lys26, exhibits high efficacy in weight control and the treatment of type II diabetes.^1–4^ This lipidation promotes self-association, which prolongs the half-life of liraglutide in blood circulation by allowing its slow release from oligomers and prevention of renal clearance.^5–7^ To ensure the reliability of liraglutide, it is pivotal to understand how its oligomerization is modulated by physiological conditions, such as temperature, ionic strength, and acidity of the solution. Traditional solution-based methods such as ultracentrifugation (AUC), small-angle X-ray scattering (SAXS), and size exclusion chromatography coupled with multiangle static light scattering (SEC-MALS) have been widely adopted to determine its oligomerization states.^6,8–11^ These studies consistently show that liraglutide forms oligomers *n* = 12–14 in mildly acid conditions (pH < 6.8) and oligomers *n* = 6–8 in neutral or slightly basic conditions (pH > 7.0), with the oligomeric transition being reversible and sensitive to pH alternations.^6^

While conventional methods are robust and widely adopted, they primarily provide ensemble-averaged measurements of oligomers in solution.^12^ This averaging can oversimplify analyte properties by emphasizing dominant oligomeric species while overlooking low-abundance intermediates, which may trigger unexpected cascading on- and off-target aggregation. For example, in neurodegenerative diseases, low-abundance prefibrillar oligomers are increasingly recognized as critical predictors of disease progression, with their modulation influencing the final fibrillation morphology.^13^ Similarly, studies on liraglutide have explored the relationship between soluble oligomers and final aggregate morphology.^6^ However, the presence of soluble oligomers failed to predict the kinetics and morphology of the final insoluble products in the current studies. This may indicate the existence of unresolved intermediate or prefibrillar oligomers that remain undefined by conventional techniques. An advanced analytical platform capable of resolving heterogeneous and low-abundance oligomers is therefore necessary to fully elucidate the oligomerization of liraglutide.

Among current analytical platforms, native mass spectrometry (nMS) has emerged as a powerful tool for resolving highly heterogeneous systems.^14–16^ Pioneering high resolution nMS platforms have resolved minute differences in masses of small molecules (i.e., lipid and metal ions) bound to membrane proteins.^17–19^ Combined with temperature-dependent experiments, high mass resolution enables the simultaneous determination of thermodynamic signatures (ΔG, ΔH, and TΔS) of up to 14 ATP binding events to the 801 kDa GroEL to study the effects of solution composition (i.e., temperature, buffer, and cofactors) in a highly heterogeneous system.^20–22^ Beyond stoichiometric information, higher-order structural information can be extracted through advanced dissociation techniques. For example, comparing *c*- and *z*-ions released from electron-capture dissociation (ECD) allows the identification of metal-binding sites, weakly bound ligands, structural modulations of proteins, and multimerization interfaces of protein complexes.^23–26^ These capabilities make nMS a unique method for studying complex biological assemblies that are difficult to resolve using solution-based techniques.

Recent advances in charge detection mass spectrometry (CDMS) and Direct Mass Technology (DMT) have further transformed mass analysis by enabling charge assignment for individual ions without relying on isotope patterns or multiple charge states.^27–31^ This advancement improves the detection of low-abundance and heterogeneous species with overlapping *m*/*z* signals, making it particularly useful for analyzing large biomolecules such as antibodies,^32,33^ spike proteins,^34^ and adeno-associated viruses (AAVs).^35–37^ These methods have been further investigated to resolve oligomer identities and structures to understand the oligomerization mechanisms. In the analysis of monoclonal antibodies (mAb), both compact and elongated oligomers with identical masses have been differentiated by two distinct charge distributions. A structural rearrangement was rationalized in pentameric and decameric mAbs, evidenced by their high abundance of compact conformers and necessitated the need for resolving conformational heterogeneity by charges.^38^ This charge-conformer relationship has been further developed as a high-throughput platform to characterize oligomers under stress conditions, resolving unusual bovine serum albumin (BSA) oligomers consisting of up to 225 monomers.^39^

Here, we present integrative mass spectrometry techniques to characterize liraglutide oligomers. ECD differentiated a restricted dynamic upon liraglutide oligomerization, which is necessary for growth to higher molecular weight (HMW) oligomers. Previously undetected oligomers of liraglutide were resolved by a direct mass technology (DMT). These experimental findings were corroborated by molecular dynamics simulations (MDS), providing explanatory mechanisms for the unusual discontinuity of oligomeric states observed in the mass domain. MDS have contributed significantly to understanding oligomerization by investigating oligomer stability,^8^ conformation,^40,41^ and self-assembly processes.^42–44^ This computational approach provides a detailed molecular basis for corroborating experimental findings.

Establishing a framework to elucidate the molecular basis of peptide oligomerization is crucial, particularly as oligomer formation can adversely affect drug potency due to reduced receptor activation.^9,45^ Additionally, a deeper understanding of oligomerization allows for the development of optimized solution conditions that enhance the physical stability of drugs and guides the rational modification and design of next-generation molecular structures. We have demonstrated a mass-spectrometry-based framework to study multiple levels in the oligomerization process and dissected the oligomerization pathways utilizing both hydrophobic and hydrophilic residues in the GLP-1 RA. This approach could add new dimensionality to the study of peptide aggregation.

## Methods

### Materials

Liraglutide (98.91% purity) was purchased from MedChemExpress LLC (Monmouth Junction, NJ). A liraglutide solution was prepared and analyzed by reconstituting the powder in 20 mM ammonium acetate to reach a concentration of 1 mg/mL without any pretreatment. The pH was adjusted by either ammonium hydroxide or acetic acid to the desired value after dissolution. The temperature of the liraglutide solution was controlled via off-line incubation in a digital temperature control incubator (Benchmark Scientific Inc., NJ) set at 37 °C unless otherwise specified. Two sample preparation strategies were applied based on the aim of the study. Time-dependent oligomerization was examined by dissolving and continuously monitoring the liraglutide samples. For ECD and DMT analyses, samples were incubated 18 h before measurement to allow for the formation of midsize (*n* = 12–18) oligomers.

### Characterization of Liraglutide Oligomers by Native Mass Spectrometry

Characterization of liraglutide oligomers was done on a Thermo Fisher UHMR system (Thermo Scientific, CA). The sample was loaded into a gold-coated pulled borosilicate ESI tip and sprayed on the Nanospray Flex Ion Source (Thermo Scientific, CA) with spray voltage set as 1.2–1.4 kV. To minimize the gas-phase activation, crucial activation energies and gas pressures were carefully examined and finalized as in-source trapping (IST): −10 V, in-source dissociation (CID): 10 V, higher energy in collision cell (HCD): 10 V, and trapping gas pressure: 5 (arbitrary unit). The resolution was set to 200,000 (*m*/*z* 400) and the *m*/*z* scan range was from 500 to 20,000 unless otherwise specified.

Direct charge assignment of liraglutide oligomers in high *m*/*z* range from 4,000 to 10,000 was done by direct mass technology (DMT) mode embedded in the Thermo Fisher UHMR system. The loading of sample and minimization of activation are identical to the above-mentioned settings except the trapping gas pressure was set to 0.2 for reduction of collision between neutral ion and gas, an essential condition for individual ion analysis.^28^ Ion filters and charge assignments were done by STORIboard build 1.0.24087.1 (Proteinaceous, IL). The modes of ion transmission and analyzer were set to high and low, respectively.

### Electron-Capture Dissociation (ECD) Mass Spectrometry

Liraglutide solution in the concentration of 1 mg/mL was sprayed on an Agilent 6545 XT Q-ToF system which was configured with a digital quadrupole (DigiQ) and an ECD cell as demonstrated in our previous work.^24^ Solutions were sprayed on a nanospray source with the electrospray voltage set to 1.5 – 1.8 kV. Wide-band isolation on the DigiQ was done by altering the duty cycle of 50.0/50.0 to 59.5/40.5 and varying the frequency value based on the q-value of 0.59. Electron-capture dissociation was done on an ExD cell (e-MSion, OR) by using an optimized set of L1-L7 DC bias and filament bias of 11 V to maximize fragmentation efficiency, as reported in our previous study.^24^ Generated spectra were deconvoluted by using MASH Native^46^ to convert isotopically resolved peaks into a list of neutral masses. The subsequent peptide mapping was done by using ClipsMS^47^ with a mass tolerance of 20 ppm error.

### Molecular Dynamics

Molecular dynamics simulations were performed using GROMACS 2023.3,^48^ utilizing the Martini 2.2 force field^49,50^ and the CHARMM36m force field.^51^ The nonstandard residue was parametrized by CHARMM-GUI^52^ and converted to course-grained beads using the CGbuilder tool.^53^ Coarse-grained structures were back-mapped to atomic structures using a backward mapping script.^54^ Liraglutide molecules were randomly placed in a periodic cubic box, ensuring a minimum separation of 2 nm. Water beads were mixed with 10% antifreeze beads as the solvent, and chloride ions were added to neutralize the system. Simulations began with energy minimization, followed by 10 ns of NVT and 10 ns of NPT equilibration. Production simulations were extended to 10 μs. Temperature was controlled by the v-rescale thermostat, and pressure was maintained at 1 bar using the Berendsen barostat for equilibration; Parrinello–Rahman barostat was used for production runs.^55,56^ The 30-mer and 45-mer simulation duplicates were conducted at 300 and 360 K, generating 8 trajectories in total. The convergence of simulated structures was examined by the radius of gyration (Rg). Simulated annealing was also conducted to evaluate structural stability and confirm dominant conformational states (see Supporting Information S7).

## Results

### Liraglutide Oligomers Characterized by Native Mass Spectrometry (nMS)

The size of liraglutide oligomers is highly dependent on solution pH.^6,9^ However, variations in oligomer sizes, such as 6-, 7-, and 8-mers in basic conditions, and 12-, 13-, and 14-mers in acidic conditions, have been reported.^6,8–10^ These discrepancies might result from the limited resolution of ensemble-based measurements, which could potentially obscure the inherent heterogeneity of the oligomeric states. Native mass spectrometry (nMS), on the other hand, provides new dimensionality for studying large molecules and resolving what is hidden with its high specificity in the mass-to-charge domain.^14^

To initiate oligomer formation, liraglutide powder was dissolved at pH 6.7 at a high concentration (1 mg/mL) and introduced into the mass spectrometer via a small orifice emitter (diameter ∼ 2 μm), preserving the oligomeric structures. While our study primarily focused on qualitative analysis, we validated the reproducibility of our mass spectrometry results by duplicating the measurements at the 30 min incubation time point, which showed similar oligomers distributions across both trials (Figure S1). In the time-course study, within 10 min of preparation, both monomeric and oligomeric forms of liraglutide were detected (see SI, Figure S2). Initially, oligomers with *n* = 2–8 (*m*/*z* 2000–3500) were more abundant than higher-order oligomers with *n* = 13–16 (*m*/*z* 3500–5000) (Figure 1a, 10 min). Prolonged incubation resulted in the formation of higher-order oligomers, with a noticeable decrease in the relative abundance of *n* = 2–8 oligomers within the *m*/*z* range of 2500–3500 (Figure 1a, 30 and 90 min). By 270 min, oligomers with *n* = 13–16 became the predominant species. After 24 h of incubation, decreased charge states in *n* = 13–16 oligomers were observed, alongside the emergence of a new oligomer (*n* = 17) primarily carrying 14 positive charges.

**Figure 1:**
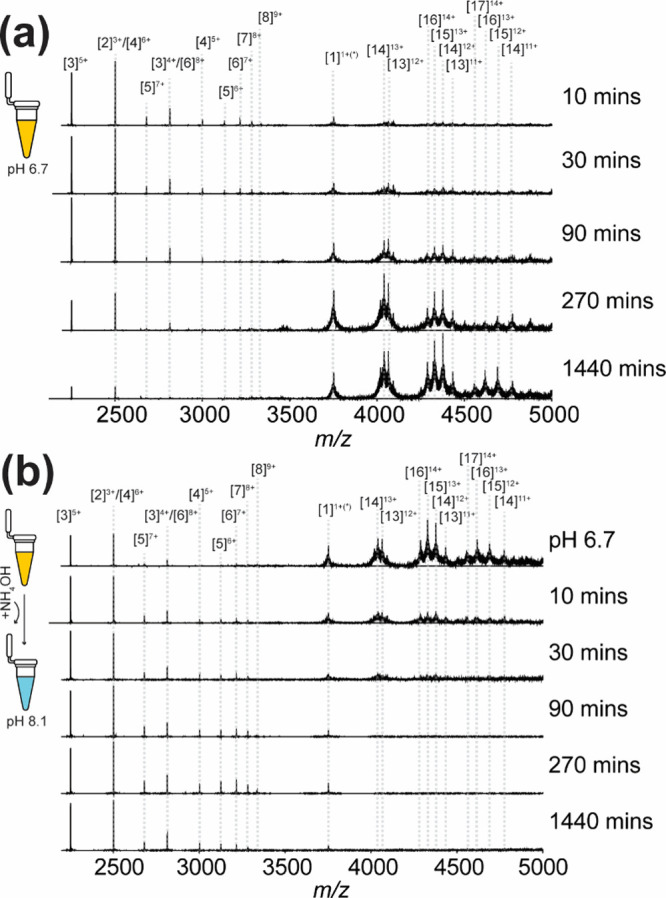
Kinetic monitoring of liraglutide oligomerization in *m*/*z* 2000–5000. (a) Liraglutide solution was incubated and monitored in 20 mM ammonium acetate (AmAc) at pH 6.7. (b) Following an 18-h incubation at pH 6.7, the solution pH was adjusted to 8.1. Oligomers are annotated as [n]^m+^, where n and m represent the oligomeric and charge state, respectively.

To evaluate the reversibility of the oligomerization process, the solution pH was increased from 6.7 to 8.1 following the formation of oligomers in the *n* = 13–17 range (Figure 1b). This pH adjustment resulted in an approximately 50% reduction in the relative abundance of these high-order oligomers within 10 min, accompanied by the rapid emergence of oligomers *n* = 2–8. These findings indicate that the dissociation of larger oligomers is required for the reformation of smaller oligomers.

The reversible transition between small and large oligomers has been attributed to the protonation state of histidine residues in liraglutide, which modulates the rate of monomer dissociation—a process identified as the rate-limiting step in oligomer transformation.^6^ Beyond the direct interconversion between two oligomeric size classes shown in previous studies, our result further revealed the simultaneous presence of multiple oligomeric states, spanning both the *n* = 3–8 and *n* = 13–17 ranges. Distinct from proteins, peptides exhibit high structural flexibility and a shallow energy landscape, allowing for a broad distribution of solution conformers.^57,58^ This structural heterogeneity may, therefore, underline the capability of liraglutide to form diverse oligomeric states.

### Electron-Capture Dissociation Identified Altered Interfacial Dynamics of Liraglutide Oligomers

We investigated the structural stability of high-order liraglutide oligomers using electron-capture dissociation (ECD). ECD is a fragmentation technique highly sensitive to the structural integrity of proteins and protein complexes and has been widely employed to detect conformational alterations induced by effectors or intermolecular interfaces.^24,59^ To enhance the detection of low-abundance *c* and *z* fragment ions, we employed an advanced digital quadrupole isolation system coupled with an ECD cell, enabling wide-band selection and efficient fragmentation.^60^

To establish a benchmark, we first isolated the 3+ liraglutide ion (*m*/*z* 1251) for ECD fragmentation (Figure 2a). Unlike conventional collision-induced dissociation (CID), ECD yields a relatively low abundance of fragment ions of ∼0.1–0.3% in relative abundance. Notably, despite isolating the 3+ ion, a 2+ ion was also detected at *m*/*z* 1876, likely resulting from gas-phase charge reduction during electron capture which has been widely noted in ECD experiments.^59^

**Figure 2:**
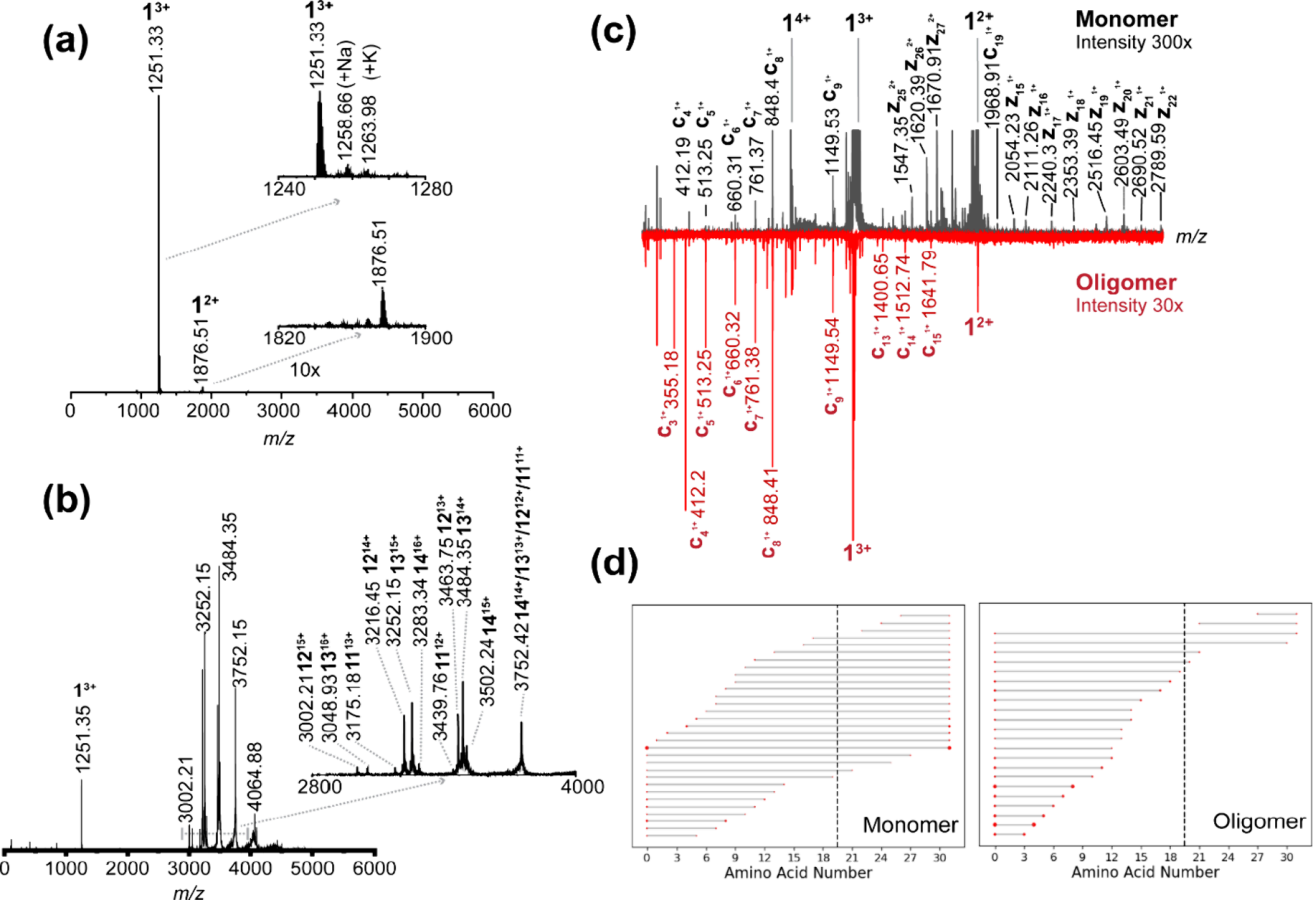
Electron-capture dissociation (ECD) mass spectrometry analysis of liraglutide monomers and oligomers. (a) ECD fragmentation of isolated monomeric liraglutide. (b) ECD fragmentation of isolated oligomeric (*n* = 12–14) liraglutide. (c) Comparison of monomeric and oligomeric liraglutide fragments in the *m*/*z* range of 350 to 3000. (d) Sequence coverage map showing identified peptide fragments of monomeric and oligomeric liraglutide. The dashed line indicates a lysine residue conjugated to palmitic acid.

To extend this analysis to oligomeric liraglutide, oligomers *n* = 11–14 (*m*/*z* 3000–4000) were isolated and fragmented (Figure 2b). Notably, despite maintaining identical solution conditions (pH 6.7, 20 mM ammonium acetate), the oligomeric distributions observed in the Q-ToF system differed slightly from those detected in the Q-Orbitrap system, with fewer oligomers preserved in the Q-ToF analysis. This discrepancy likely arises from suboptimal ion transmission in the current Q-ToF configuration. Although such variations highlight potential differences arising from activation parameters or instrument design, both mass spectrometers consistently revealed heterogeneous oligomeric states that were previously unresolved by conventional analytical methods. These findings underscore the power of mass spectrometry in resolving highly complex oligomeric assemblies within a single analysis.

Fragmentation analysis revealed that both monomeric and oligomeric liraglutide primarily generated singly or doubly charged *c*- and *z*-ions distributed across the *m*/*z* range of 400–2700 (Figure 2c). Notably, the fragmentation efficiency was higher in oligomeric liraglutide due to the higher charge states facilitating the electron-capture process. However, in the *m*/*z* 2000–2700 range, a reduction in *z*-ions was observed for oligomeric liraglutide (Figure 2c, bottom panel).

To correlate these fragmentation patterns with structural features, we mapped the product ions onto a sequence coverage map of liraglutide (Figure 2d). The coverage map demonstrates that oligomeric liraglutide yielded fewer *c*- and *z*-fragments than monomeric liraglutide, with 30 and 23 *c*, *z*-ions identified for monomeric and oligomeric liraglutide, respectively. Of these fragments, 18 and 4 *c*, *z*-ions containing palmitic acid conjugates were detected in the monomeric and oligomeric forms, respectively. This difference aligns with the proposed role of the palmitic acid conjugate as a hydrophobic motif driving oligomerization. In addition, a significant difference was also observed in the number of *z*-ions released. Whereas only 2 *z*-ions were released from the oligomer, 18 *z*-ions were released from the monomer (Figure 2d). The reduced number of ions observed in ECD has often been interpreted as revealing both flexible (exposed) and protected (buried) sites in protein complexes and oligomers.^24,61–63^ Hence, the lower number of ions observed in oligomeric liraglutide suggests the protection of its palmitic acid conjugating site and the restriction of the dynamics in its C-terminal residues.

### Newly Discovered Higher Oligomeric States of Liraglutide Observed by Direct Mass Technology Measurement

In addition to the previously reported soluble oligomers, we detected low-abundance species in the *m*/*z* range of 5,000–10,000 after an 18-h incubation in 20 mM ammonium acetate (pH 6.7). However, the overlapping signals within this range produced low-resolution spectra with convoluted peak distributions, precluding accurate charge state determination^64^ using conventional charge state distribution analysis (Figure 3a). This limitation was overcome by using Direct Mass Technology (DMT), a method in which the accumulation of ion signal intensity over analysis time is proportional to the ion charge,^27,28,65^ enabling charge assignment by analyzing the slope of summed signal intensity versus analysis time.

**Figure 3:**
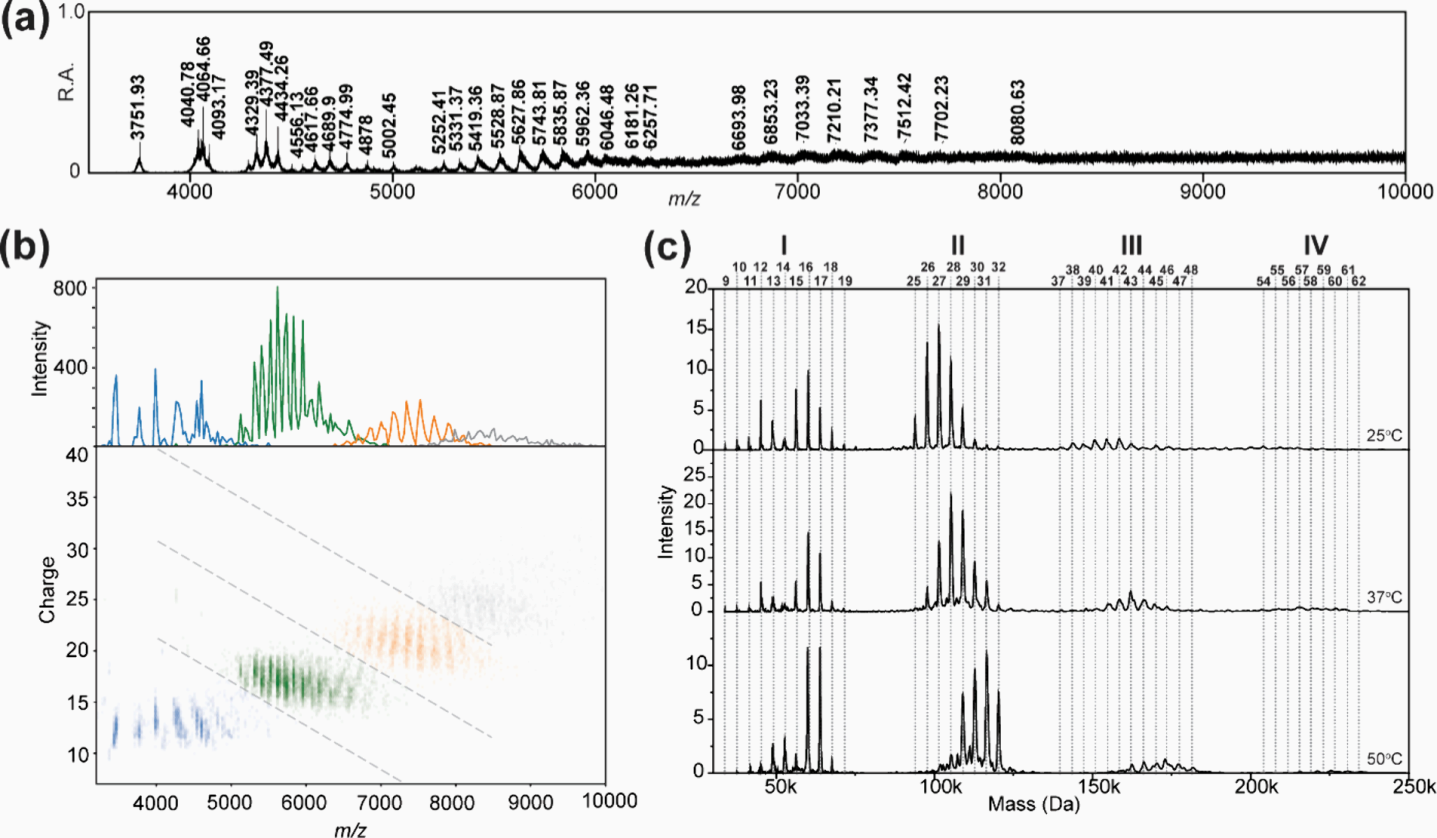
Liraglutide oligomers in the high mass range. (a) Mass spectra were acquired in conventional MS mode. (b) Two-dimensional (2D) heatmap analysis of data acquired in the DMT mode. The charge state of individual ions was assigned using STORI analysis. Clusters are color-coded based on the distinct regions (I–IV) observed in (c). (c) Direct mass spectra showing four distinct regions (I–IV) at different incubation temperatures. Oligomeric states are indicated at the top of the spectra.

Using DMT, we determined that the charge states of these high *m*/*z* species primarily ranged from 8+ to 75+ (Figure 3b). Crucially, we found that optimizing charge assignment parameters, such as narrowing the bin size and reducing the minimum number of ions required for analysis, was essential to retaining sufficient ions for subsequent charge assignments. This was not achievable using the default STORI charge assignment parameters (SI Figure S3). Visualization of *m*/*z* (*x*-axis) versus charge (*y*-axis) revealed four distinct charge-state clusters, which were color-coded in the raw spectra for validation (Figure 3b). Mapping these color assignments back to their respective *m*/*z* domains clearly delineated overlapping signal regions, highlighting the complexity of charge distribution in this *m*/*z* range (Figure 3b, orange, and gray). This charge assignment enabled mass determination of the newly identified oligomers, which ranged from ∼100 to 250 kDa, corresponding to oligomeric states of *n* = 25–62 (Figure 3c).

Interestingly, rather than forming a continuous distribution of oligomeric states from *n* = 12 to *n* = 62, these high molecular weight (HMW) oligomers segregated into four distinct clusters: *n* = 9–19 (Region I), *n* = 25–32 (Region II), *n* = 37–48 (Region III), and *n* = 54–62 (Region IV) (Figure 3c). To investigate the influence of solution conditions on the distribution of these HMW oligomers, we evaluated the effects of the pH and temperature. While HMW oligomers were undetectable at elevated pH (SI Figure S4), increasing the temperature at pH 6.7 promoted oligomer growth across all four regions (Figure 3c). In Region I, a bimodal distribution emerged, with two dominant states (*n* = 12 and *n* = 16) at 25 °C. At higher temperatures (37 and 50 °C), the distribution became more uniform, with *n* = 16 as the dominant species. Notably, this bimodal distribution was exclusive to Region I, whereas in the remaining three regions, elevated temperatures led to a general shift toward higher oligomeric states. The distinct segregation of oligomeric clusters and the unusual bimodal distribution in Region I prompted further investigation via molecular dynamics simulations to elucidate the underlying mechanisms driving these structural phenomena.

### Molecular Dynamics Simulation Proposed a Plausible Oligomerization Mechanism Coherent with DMT Observation

To elucidate the formation of high-mass oligomers observed in our experiments, we performed a 10-μs coarse-grained molecular dynamics (MD) simulation with 30 and 45 liraglutide monomers. The monomers were initially randomized within a confined 26.22 (for 30-mer) or 29.20 (for 45-mer) nm^3^ box with explicit water molecules (liraglutide concentration ∼ 3 mM) and assigned charges based on their propensity to carry charges at pH 6.7.

As an amphiphilic peptide,^66^ liraglutide undergoes self-assembly driven by a balance between hydrophobic and hydrophilic interactions. To determine their individual contributions to oligomerization, predefined hydrophobic and hydrophilic residues were color-mapped in red (hydrophobic) and blue (hydrophilic) (Figure 4a). At 0 μs, each monomer exhibited a distinct segregation of hydrophobic and hydrophilic regions, primarily attributed to the hydrophobicity of D6M and the hydrophilicity of the backbone residues (Figure 4a, 0 μs). As the simulation progressed, liraglutide monomers associated into small, transient oligomers (*n* = 5–8) via hydrophobic interactions (Figure 4a, 0.4 μs). These oligomers evolved into larger oligomers through the fusion of two oligomers. Interestingly, unlike the assembly process of small oligomer formation, the generation of large oligomers (*n* = 12–15) required structural rearrangement of the micelle-like core, as evidenced by the presence of dispersed hydrophobic cores which then converged to defined hydrophobic cores (Figure 4a, 3 μs). By the end of the simulation (10 μs), two distinct clusters had formed, exhibiting a clear hydrophilic interface and separated dense hydrophobic centers. Collectively, the growth of large oligomers ceased once a critical size was reached, at which point *n* = 12–15 oligomers acted as building blocks for higher order assemblies driven by hydrophilic interactions. MD simulations provide a mechanistic explanation for the discontinued oligomeric states observed beyond *n* = 19 and the origins of the higher-order oligomers (*n* = 25–32, 37–48, and 54–62) identified by our direct mass measurements.

**Figure 4:**
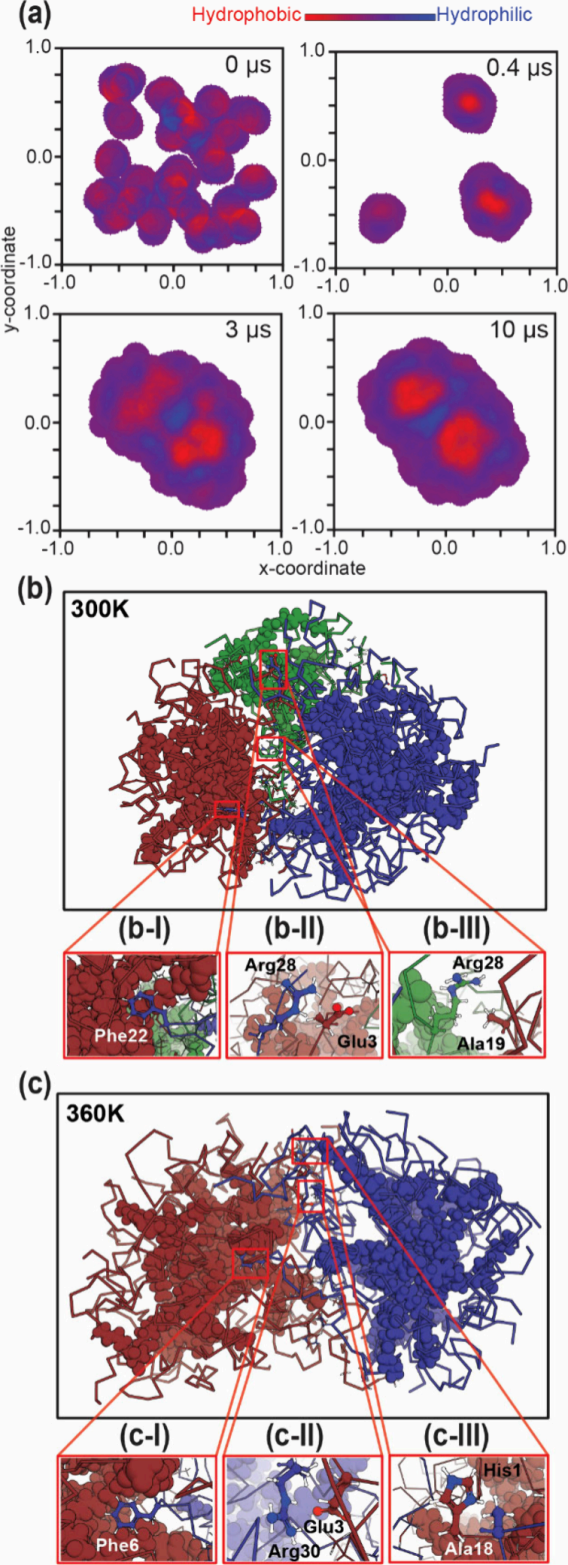
Representative frames and structures from molecular dynamics simulations of liraglutide self-assembly. (a) Snapshots at 0, 0.4, 3 , and 10 μs show the assembly process of 30 liraglutide monomers. Hydrophobic and hydrophilic regions are labeled in red and blue, respectively. Structural comparison of the 30-mer at (b) = 300 K and (c) = 360 K (10 μs). Peptide backbones (ribbon) and conjugated palmitic acid motifs (spheres) are shown. The number of clusters, determined by the number of dense hydrophobic cores shown in (a), is visualized using green, red, and blue: three clusters at (b) 300 K and two at (c) 360 K. Representative residue pair interactions (b-I, b-II, b-III and c-I, c-II, c-III) are shown in the insets.

A detailed contribution of individual residues was analyzed to investigate the molecular forces stabilizing these assemblies (Figure 4b). Three types of interaction pairs were classified: hydrophobic, hydrophilic, and hybrid. For example, van der Waals interactions between phenylalanine and the palmitic acid tail were categorized as hydrophobic pair interactions (Figure 4, b-I & c-I). Analogously, the short distance (<4.5Å) between arginine and glutamic acid (Figure 4, b-II&c-II) was recognized and defined as hydrophilic pair interactions. The final category, hybrid pairs, accounted for interactions between polar and nonpolar residues, which were predominantly stabilized by dipole–induced dipole interactions (Figure 4, b-III&c-III). Unlike the process observed for small (*n* = 2–8) and large oligomers (*n* = 12–18), in these high molecular weight oligomers, analysis of interfacial interactions revealed a strong contribution from 32 to 61% hydrophilic–hydrophilic and 20–55% hybrid interactions (Table S3). Residue analysis alongside the absence of hydrophobic core rearrangement during the assembly of high-order oligomers suggested that electrostatic and hydrogen bond networks on the surface are the primary driving forces in their association.

As the simulation corroborated our DMT findings, we extended this approach to investigate the effects of the temperature. In addition to the baseline simulation at 300 K (Figure 4b), we conducted additional simulations at 360 K (Figure 4c) while maintaining all other parameters constant. Simulation at each temperature was performed in duplicate to ensure the reliability of our observations. By the end of the simulations, all 30 liraglutide monomers had assembled into a large 30-mer, with minor variations in intermediate oligomeric states (Figure S5). Notably, at 300 K, two and three distinct clusters formed, with oligomeric sizes of 8–22 and 5–10–15 (where *x*–*y*–*z* denotes the number of liraglutide molecules in each cluster). At 360 K, the simulations yielded two clusters with oligomeric sizes of 12–18 and 13–17. Extended study using 45 liraglutide monomers showed the clusters of 9–10–11–15, 5–6–14–20 and 8–9–10–18, 10–17–18 at 300 and 360 K, respectively (Table S4). These results highlighted that the increase of temperature yielded a more uniform distribution of the large oligomers as building blocks. Detailed examination of the course of simulation showed that elevated temperatures promote the growth of *n* = 9–18 oligomers into larger assemblies by increasing peptide backbone flexibility and facilitating structural rearrangement. Additionally, high temperature promoted an increased frequency of the formation and disruption of interfacial contacts, enabling oligomers with suboptimal interactions (high-energy states) to dissociate and reassociate into a more stable oligomeric form. This observation offered a mechanistic explanation for our DMT data, which showed a more uniform oligomer distribution at higher temperatures (37 and 50 °C) compared to 25 °C in Region I (Figure 3c, *n* = 9–18). Subsequently, the increased size of these *n* = 9–18 building block oligomers at elevated temperatures contributes to the overall growth of high-order oligomers (*n* = 25–32, 37–48, and *n* = 54–62) through the previously observed hydrophilic interactions, resulting in the observed increase of oligomeric states in Region II–IV detected by DMT.

## Discussion and Conclusion

Conjugating therapeutic peptides to generate amphiphilic properties has promoted successful drug delivery, enhanced pharmacokinetics, and increased stability in the human circulatory system.^67–69^ However, the propensity for oligomerization can induce insoluble aggregates, adversely affecting both the manufacturing process and the biomedical function. The formation of insoluble species has been identified by transmission electron microscopy forming micrometer-scale fibrillar or amorphous aggregates.^9^ While identification of these oligomers is crucial since they are potential precursors or inhibitors of the final precipitated insoluble products,^70^ stress studies failed to correlate the morphology of insoluble aggregates to the composition of prefibrillation oligomers,^9^ likely due to the limited resolution and sensitivity of conventional solution-based methods. Native mass spectrometry (nMS) offers a highly specific approach analyzing hidden soluble oligomers and deciphering the underlying mechanisms of their formation. Advancements in fragmentation and detection in native mass spectrometry broaden the scope of oligomer analysis, including identification of the oligomers’ interfacial dynamics and the exploration of previously hidden species.

Native mass spectrometry can resolve highly heterogeneous systems to reveal detailed insights across a wide range of mass domains. At *m*/*z* ranges less than 3000, corresponding to oligomeric states *n* = 2 to 8 of liraglutide, masses were unambiguously determined by isotopically resolved signals offered by high-resolution Orbitrap mass spectrometry. In *m*/*z* 3000 to 5000, oligomers identities were resolved based on their charge state distribution (CSD). However, high heterogeneity and low abundance of species posed challenges to confident determination of species beyond *m*/*z* 5000. This challenge was addressed by extending the analysis from conventional MS to the direct mass technology (DMT) mode, enabling direct assignment of charges to individual ions followed by mass determination. This unambiguous assignment of oligomers allowed us to monitor the assembly trajectory during the course of incubation. On top of the oligomers from *n* = 2 to *n* = 17, novel liraglutide oligomers *n* = 25 to 62 shown in DMT directed a new oligomerization mechanism distinct from that involving hydrophobic conjugates.

Combining electron-capture dissociation (ECD) and molecular dynamics simulations (MDS) provided mechanistic insights into the oligomerization pathway following the formation of oligomers with *n* = 12–18. ECD was used to probe the structural dynamics of these oligomers, while MDS provided a detailed view of their interactions and assembly. The decreased release of *z*-ions observed after the formation of 12- to 14-mers was interpreted as an indication of restricted C-terminal residue dynamics in larger oligomers. Based on ECD and MDS results, we propose two essential processes for the evolution of *n* = 12–18 oligomers into higher-order (*n* = 25–62) oligomers: (i) ECD results suggested restriction of C-terminal residues stabilize the structures of *n* = 12–18 oligomers and (ii) MDS provided evidence that subsequent association with hydrophilic residues leads to the formation of segregated clusters, as detected by direct mass spectrometry. The insights provided by simulation results promoted us to conduct a comprehensive examination of the liraglutide oligomerization process with a focus on the conformational and energy landscapes adopted during oligomerization. We have presented these findings in a separate paper submitted in parallel with this paper.^200^

Identifying the intermediate oligomers is challenging and requires advanced analytical platforms. Distinct from the folded protein in the biological system, the inherent flexibility of peptide backbones allows peptides to adopt a wide range of conformers. The conjugation of a hydrophobic moiety further diversifies the conformational landscape of peptide therapeutics. Uncertainty regarding conformational distribution can cascade off-pathway oligomerization and eventually insoluble aggregates. Although dual oligomerization pathways are commonly described in controlling the morphology of nanomaterials formed from amphiphilic building blocks,^71–73^ studies explicitly noting this phenomenon in the context of therapeutic conjugates are limited. While current studies extensively focus on oligomers formed via hydrophobicity imparted by conjugated lipid tails, we identified higher-order oligomers formed through a combination of hydrophobic and hydrophilic interactions. The presence of these higher-order oligomers may be more indicative of fibrillation observed by microscopic analysis.^74,75^ Parallel to other analytical techniques, we have demonstrated a promising array of mass spectrometric techniques to identify uncommon oligomers in solution, supported by simulation data. We hope this will provide a novel framework for the discovery of unexpected high-order oligomers, which may risk aggregation or reduced efficacy, in the fields of drug discovery, design, and development.

## Supplementary Material



## Notes

Full range mass spectra, optimization of direct mass charge assignment parameters, analysis of high-order oligomers at high pH condition, molecular dynamics process of high-order oligomers, summary of structures of liraglutide oligomers analyzed by MD (PDF)
