# Supplementary material for: Dissecting Hidden Liraglutide Oligomerization Pathways via Direct Mass Technology, Electron-Capture Dissociation, and Molecular Dynamics
Source: Anal Chem. 2025 Jun 16;97(25):13465–73. doi: 10.1021/acs.analchem.5c01851 (PMC12224166; doi:10.1021/acs.analchem.5c01851)
Supplement: Supplementary file 1 [file ac5c01851_si_001.pdf]

SUPPORTING INFORMATION

Dissecting Hidden Liraglutide Oligomerization Pathways via Direct Mass Technology, Electron-Capture Dissociation, and Molecular Dynamics

Syuan-Ting Kuo[1], Zhenyu Xi[1], Xiao Cong[2], Xin Yan[1], and David H. Russell[1]
[1]Department of Chemistry, Texas A&M University, College Station, Texas 77843, USA
[2]Boehringer Ingelheim, Ridgefield, Connecticut, 06877, USA

Table of Contents

	Page
S1. Mass Spectra Replicates of Liraglutide Oligomers	
Figure S1	S2
S2. Full Mass Range Analysis of Liraglutide Oligomers	
Figure S2	S3
Table S1	S3
S3. Optimization of Charge Assignment Parameters for Liraglutide Analysis	
Table S2	S4
Figure S3	S5
S4. Analysis of High-Order Oligomers at pH 6.7 and 8.1	
Figure S4	S6
S5. Molecular Dynamics Simulation of 30 Liraglutide Monomer Assembly	
Figure S5	S6
S6. Interaction Network of High-Order Oligomers from Molecular Dynamics Simulations	
Table S3	S7
Table S4	S9
S7. Enhanced Sampling to Evaluate the Convergence of Simulation Process and Stability of Conformers	
Figure S6	S17
Table S5	S18
Reference	S18

S1. Mass Spectra Replicates of Liraglutide Oligomers

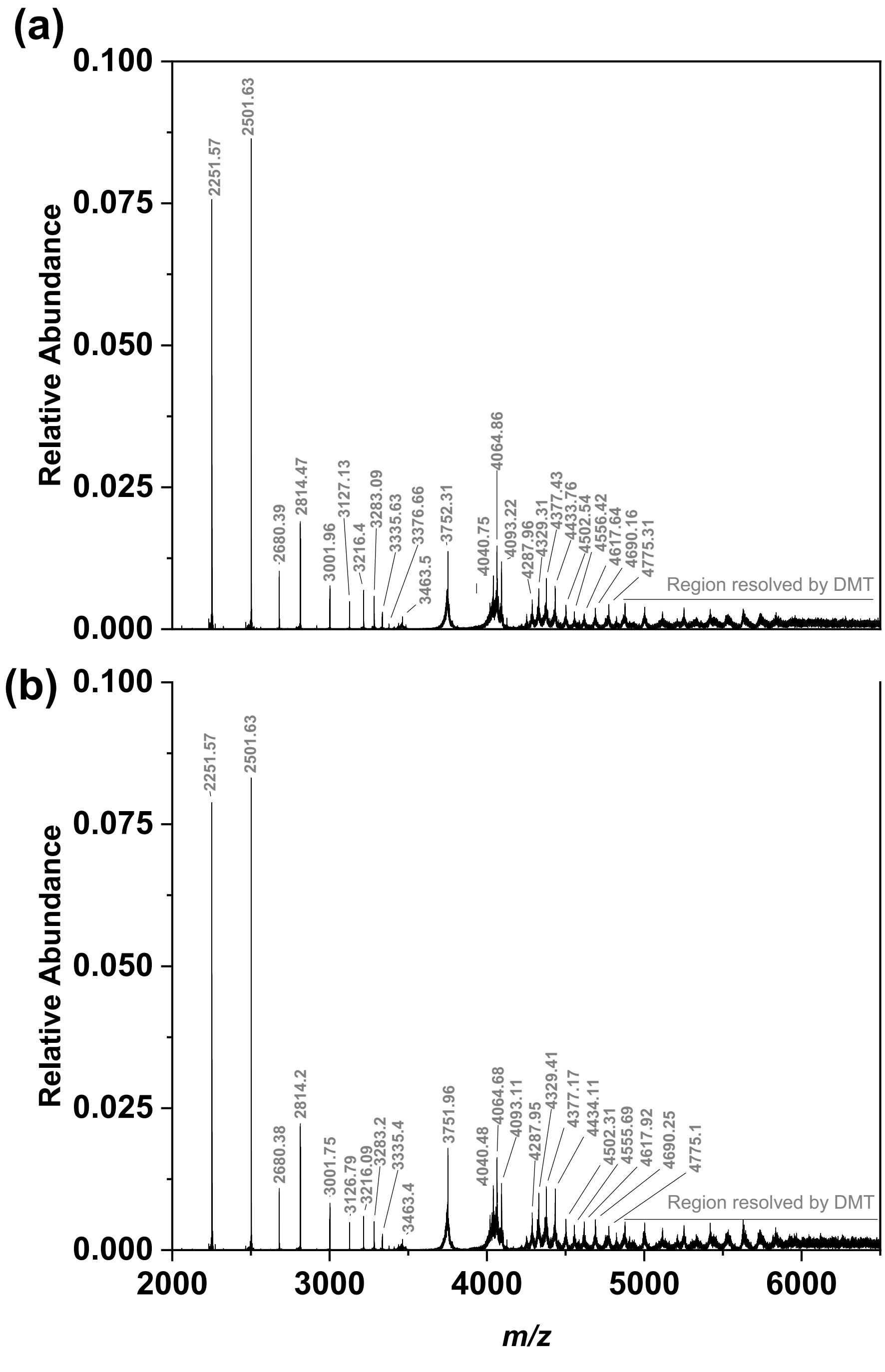

Figure S1. Replication of liraglutide oligomer mass spectrometry measurements. Panels (a) and (b) show biological replicates acquired at a single 30-minute time point.

S2. Full Mass Range Analysis of Liraglutide Oligomers

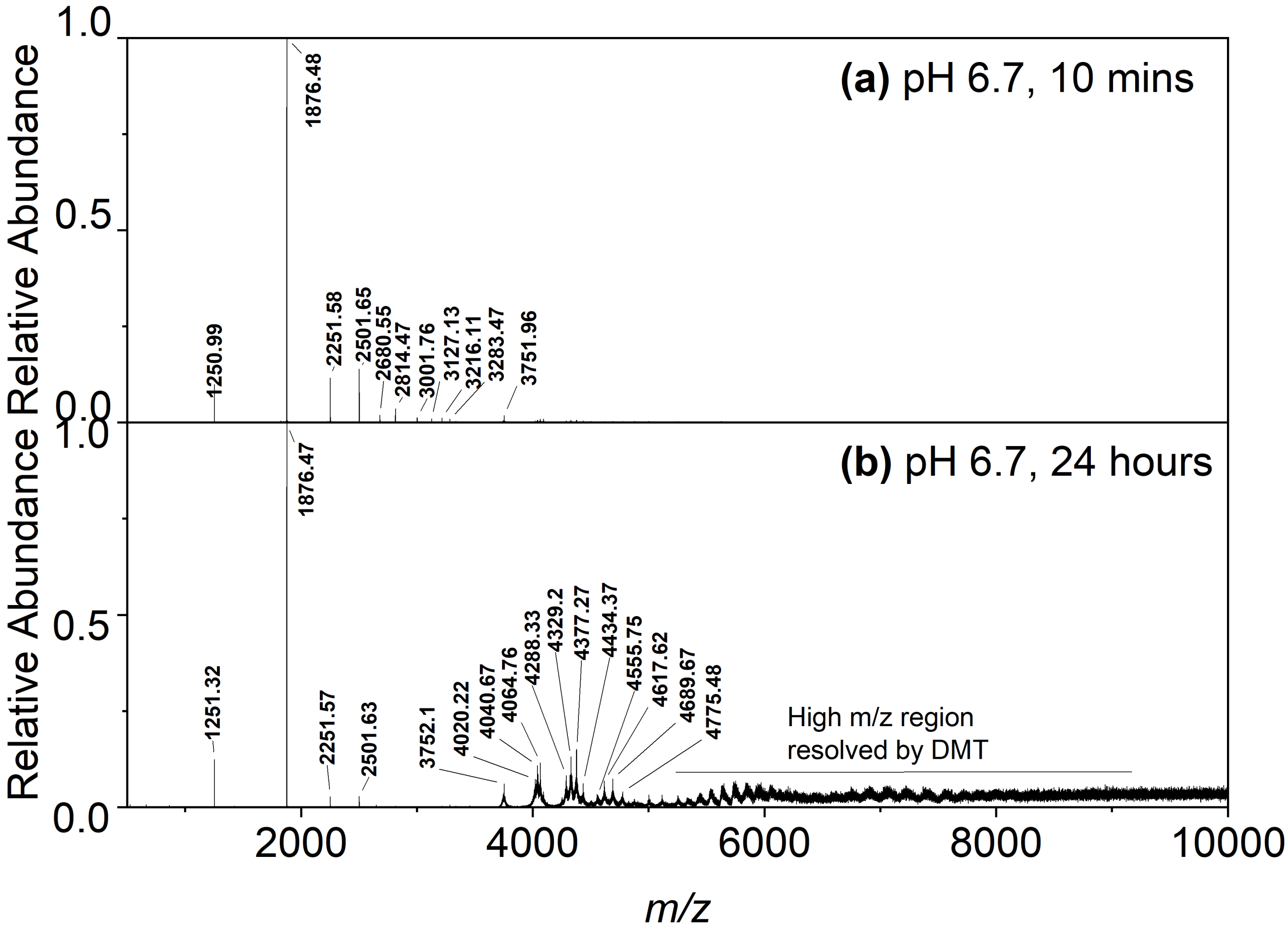

Figure S2. Full mass range (*m/z* 500-10000) of mass spectra of liraglutide in the solution at pH 6.7 after (a) 10 min and (b) 24 h. Liraglutide masses were determined from isotopically resolved peaks or charge state distribution and summarized in **Table S1**.

Table S1. Masses, charge, and oligomeric states of liraglutide oligomers determined by isotopically resolved peaks or charge state distribution.

m/z	Charge	Mass (Da)	Oligomeric States (n)	Deviation (Da)	Charge Determination
1250.99	3	3749.958	1	-1.24	Isotopic distribution
1876.48	2	3750.952	1	-0.25	Isotopic distribution
2251.58	5	11252.88	3	-0.73	Isotopic distribution
2501.65	3	7501.938	2	-0.47	Isotopic distribution
2680.55	7	18756.822	5	0.81	Isotopic distribution
2814.47	8	22507.728	6	0.52	Isotopic distribution
3001.76	5	15003.78	4	-1.03	Isotopic distribution
3127.13	6	18756.756	5	0.75	Isotopic distribution
3216.11	7	22505.742	6	-1.47	Isotopic distribution

3283.47	8	26259.728	7	1.31	Isotopic distribution
3751.96	1/14	3750.956/ 52513.384	1/14	0.75/-3.44	Charge state distribution
4020.22	14	56269.024	15	0.99	Charge state distribution
4040.67	13	52515.658	14	-1.17	Charge state distribution
4064.76	12	48765.072	13	-0.55	Charge state distribution
4288.33	14	60022.564	16	3.33	Charge state distribution
4329.2	13	56266.548	15	-1.48	Charge state distribution
4377.27	12	52515.192	14	-1.64	Charge state distribution
4434.37	11	48767.026	13	1.4	Charge state distribution
4555.75	14	63766.444	17	-3.99	Charge state distribution
4617.62	13	60016.008	16	-3.22	Charge state distribution
4689.67	12	56263.992	15	-4.04	Charge state distribution
4775.48	11	52519.236	14	2.41	Charge state distribution

S3. Optimization of Charge Assignment Parameters for Liraglutide Analysis

Table S2. Optimization of charge assignment parameters in the STORI voting v3 charge assigner. (a) Default parameters with a wide bin size resulted in excessive ion filtering. (b) Narrowing the bin size reduced ion filtering, while adjusting the minimum number of ions per bin compensated for the reduced number of ions in each bin. A total of 2,702,223 ions were analyzed and filtered using both STORI processor and charge assignment parameters to minimize false positive identifications.

	(a) Default	(b) Narrow window
Parameter		
Bin size (ppm)	1.5	1
Minimum Ions in Bin	2	1
Numbers of Charge Neighbors	2	2
Number of Isotope Neighbors	10	10
Result		
Assigned ion counts	389	11,290
Assigned charge range	11-19	8-75

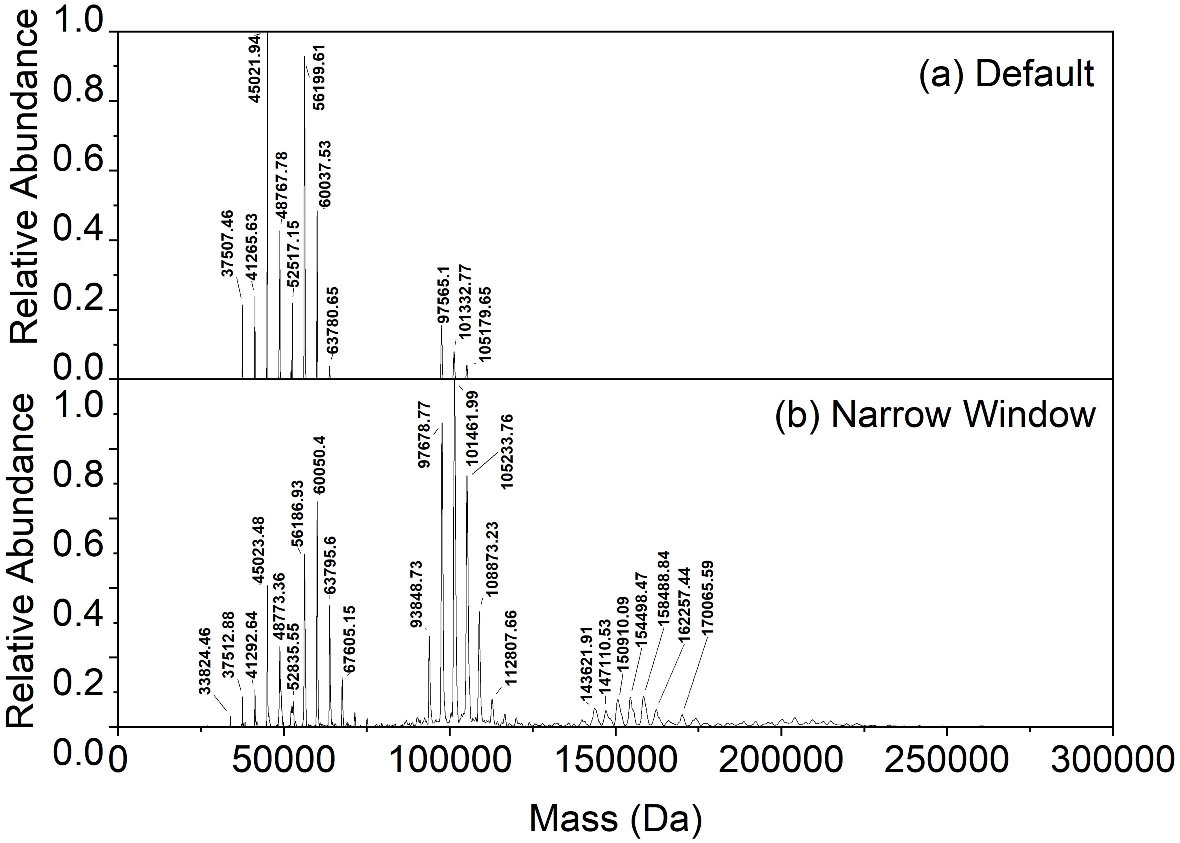

Figure S3. Direct mass spectra of liraglutide at pH 6.7 and 25 °C resulting from the charge assignment using (a) default and (b) narrow bin size windows

S4. Analysis of High-Order Oligomers at pH 6.7 and 8.1

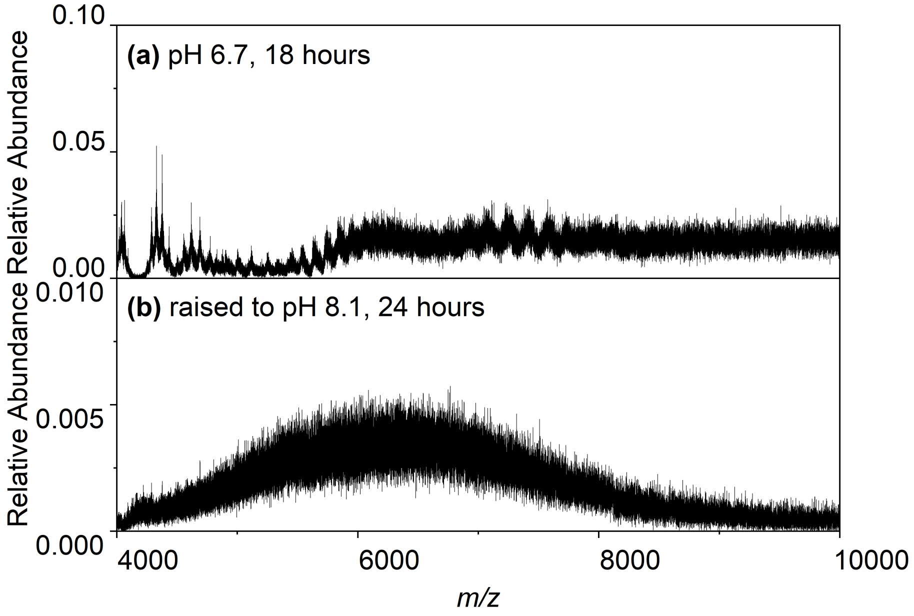

Figure S4. Zoomed mass spectra (m/z 4,000-10,000) of liraglutide in solution at (a) pH 6.7, incubated 18 h (initial condition) and (b) pH 8.1 after 24 h of incubation.

S5. Molecular Dynamics Simulation of 30 Liraglutide Monomer Assembly

Figure S5. Molecular dynamic simulation of 30 liraglutide molecules assembling at 300K. Hydrophobic and hydrophilic regions are shown in red and blue, respectively.

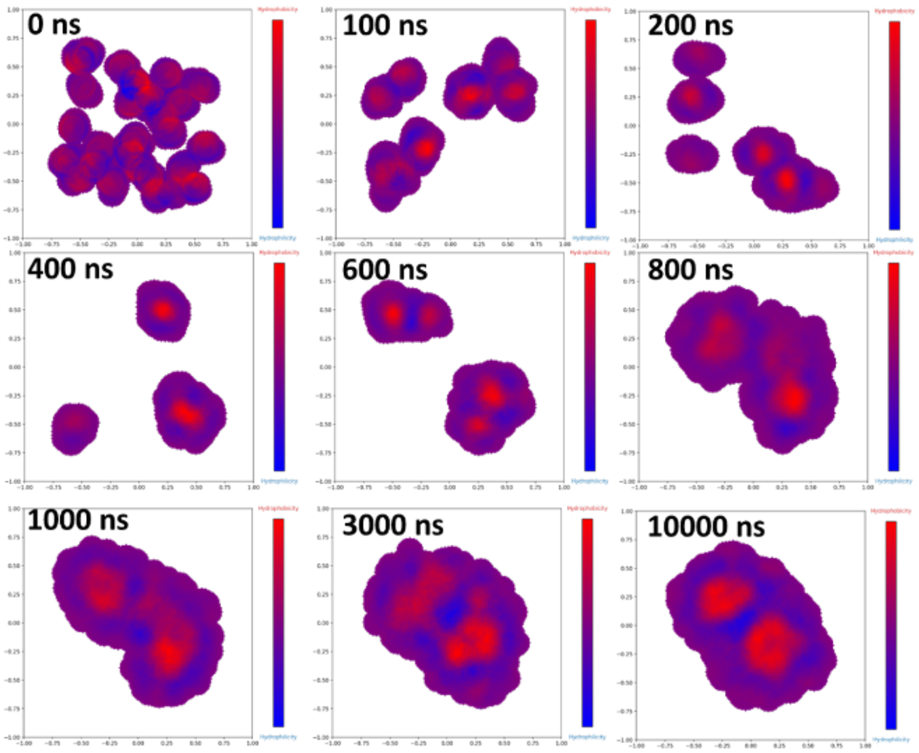

S6. Interaction Network of High-Order Oligomers from Molecular Dynamics Simulations

Table S3. Summary of end-point structure from eight simulation results. Subunit composition indicates the number of liraglutide monomers in each subunit. Contact contributions were calculated from residue statistics presented in **Table S4**.

Condition	Structure	Hydrophobic Contacts Contribution (%)	Hydrophilic Contacts Contribution (%)	Hybrid Contacts Contribution (%)	Subunit Composition (w-x-y-z)	Averaged Subunit Size (n)
30mer-300K-Rep1	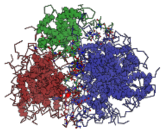	22	32	46	5-10-15	10 ± 5
30mer-300K-Rep2	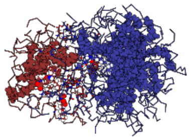	22	35	43	8-22	15 ± 10
30mer-360K-Rep1	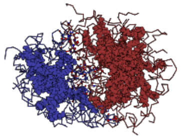	6	61	33	12-18	15 ± 4
30mer-360K-Rep2	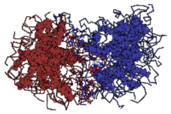	27	53	20	13-17	15 ± 3
45mer-300K-Rep1	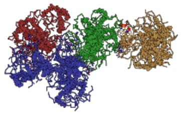	19	36	45	9-10-11-15	11 ± 3

45mer-300K-Rep2	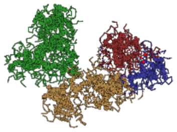	16	55	29	5-6-14-20	11 ± 7
45mer-360K-Rep1	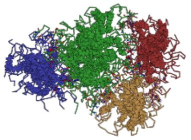	10	35	55	8-9-10-18	11 ± 7
45mer-360K-Rep2	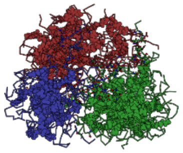	12	47	41	10-17-18	15 ± 4

Table S4. Interfacial residue pairs in **(a)** 30-mer, 300K, replicate 1

Entry	Cluster Pair	Chain 1	Res1#	Res1 Name	Chain 2	Res2#	Res2 Name	Distance (Å)
1	1-2	J	10	VAL	F	24	ALA	4.11
2	1-2	J	11	SER	F	30	ARG	4.41
3	1-2	K	12	SER	d	5	THR	4.39
4	1-2	T	24	ALA	D	11	SER	4.46
5	1-2	Y	10	VAL	N	4	GLY	3.94
6	1-2	Y	11	SER	N	4	GLY	3.87
7	1-2	Y	15	GLU	N	1	HIS	4.47
8	1-2	Y	18	ALA	D	5	THR	3.58
9	1-2	Y	19	ALA	D	18	ALA	3.97
10	1-2	Y	24	ALA	N	5	THR	4.49
11	1-2	Y	24	ALA	N	10	VAL	3.98
12	1-2	a	4	GLY	F	30	ARG	4.50
13	1-2	a	8	SER	F	27	VAL	4.44
14	1-2	a	9	ASP	d	7	THR	3.86
15	1-2	b	24	ALA	D	10	VAL	4.40
16	1-2	b	29	GLY	D	8	SER	3.75
17	1-3	J	28	ARG	X	3	GLU	4.28
18	1-3	J	29	GLY	X	5	THR	4.41
19	1-3	L	17	GLN	M	14	LEU	4.47
20	1-3	L	19	ALA	M	11	SER	4.32
21	1-3	R	2	ALA	P	3	GLU	4.34
22	1-3	R	2	ALA	P	4	GLY	3.81
23	1-3	T	10	VAL	W	7	THR	4.03
24	1-3	T	18	ALA	M	19	ALA	4.20
25	1-3	T	22	PHE	M	20	D6M	1.95
26	1-3	T	30	ARG	W	4	GLY	3.97
27	1-3	b	7	THR	P	2	ALA	3.82
28	1-3	c	9	ASP	M	2	ALA	4.19
29	1-3	c	11	SER	M	2	ALA	4.04
30	2-3	D	9	ASP	M	1	HIS	4.05
31	2-3	D	9	ASP	M	10	VAL	3.89
32	2-3	D	14	LEU	P	10	VAL	4.49
33	2-3	D	28	ARG	E	19	ALA	4.27
34	2-3	N	2	ALA	X	7	THR	4.17
35	2-3	N	16	GLY	U	3	GLU	3.78
36	2-3	N	19	ALA	O	19	ALA	3.75
37	2-3	V	9	ASP	E	24	ALA	3.87

(b) 30-mer, 300K, replicate 2

Entry	Cluster Pair	Chain 1	Res1#	Res1 Name	Chain 2	Res2#	Res2 Name	Distance (Å)
1	1-2	B	5	THR	a	4	GLY	3.94
2	1-2	G	2	ALA	J	3	GLU	4.37
3	1-2	G	2	ALA	J	5	THR	3.65

4	1-2	G	3	GLU	J	2	ALA	3.89
5	1-2	G	8	SER	K	2	ALA	3.49
6	1-2	N	7	THR	c	21	GLU	4.39
7	1-2	N	8	SER	c	24	ALA	3.89
8	1-2	N	10	VAL	c	24	ALA	4.19
9	1-2	N	11	SER	c	29	GLY	3.59
10	1-2	N	20	D6M	R	13	TYR	4.26
11	1-2	Q	27	VAL	c	24	ALA	4.23
12	1-2	S	8	SER	P	20	D6M	4.49
13	1-2	S	13	TYR	V	20	D6M	3.75
14	1-2	S	15	GLU	R	2	ALA	3.89
15	1-2	S	16	GLY	R	2	ALA	3.70
16	1-2	S	16	GLY	R	5	THR	3.58
17	1-2	S	20	D6M	R	10	VAL	1.66
18	1-2	T	4	GLY	J	1	HIS	4.32
19	1-2	T	12	SER	V	30	ARG	4.12
20	1-2	U	7	THR	R	3	GLU	4.38
21	1-2	U	7	THR	R	4	GLY	4.24
22	1-2	Z	4	GLY	P	19	ALA	4.01
23	1-2	Z	11	SER	P	24	ALA	3.88

(c) 30-mer, 360K, replicate 1

Entry	Cluster Pair	Chain 1	Res1#	Res1 Name	Chain 2	Res2#	Res2 Name	Distance (Å)
1	1-2	B	4	GLY	Z	8	SER	4.45
2	1-2	B	4	GLY	Z	9	ASP	4.04
3	1-2	B	29	GLY	Z	7	THR	3.89
4	1-2	E	12	SER	P	25	TRP	4.27
5	1-2	E	15	GLU	P	30	ARG	4.46
6	1-2	G	2	ALA	H	3	GLU	3.60
7	1-2	G	2	ALA	a	4	GLY	3.54
8	1-2	G	12	SER	P	8	SER	4.16
9	1-2	L	7	THR	T	29	GLY	4.40
10	1-2	L	15	GLU	A	4	GLY	3.86
11	1-2	L	17	GLN	A	2	ALA	3.99
12	1-2	O	2	ALA	P	4	GLY	4.34
13	1-2	O	4	GLY	P	4	GLY	4.41
14	1-2	O	10	VAL	P	1	HIS	3.86
15	1-2	O	27	VAL	a	10	VAL	4.12
16	1-2	O	28	ARG	a	7	THR	4.47
17	1-2	b	7	THR	C	31	GLY	4.22

18	1-2	b	8	SER	C	31	GLY	3.83

(d) 30-mer, 360K, replicate 2

Entry	Cluster Pair	Chain 1	Res1#	Res1 Name	Chain 2	Res2#	Res2 Name	Distance (Å)
1	1-2	C	1	HIS	I	15	GLU	3.94
2	1-2	C	11	SER	I	10	VAL	4.34
3	1-2	C	24	ALA	a	23	ILE	3.87
4	1-2	J	1	HIS	I	31	GLY	4.45
5	1-2	J	4	GLY	I	28	ARG	4.19
6	1-2	J	10	VAL	a	29	GLY	4.40
7	1-2	Q	7	THR	W	18	ALA	4.00
8	1-2	Q	20	D6M	I	6	PHE	3.51
9	1-2	X	24	ALA	a	14	LEU	3.93
10	1-2	c	1	HIS	H	11	SER	4.13
11	1-2	c	1	HIS	H	12	SER	3.84
12	1-2	c	2	ALA	T	18	ALA	3.95
13	1-2	c	3	GLU	T	30	ARG	3.53
14	1-2	c	5	THR	H	1	HIS	4.41
15	1-2	c	8	SER	T	15	GLU	3.64

(e) 45-mer, 300K, replicate 1

Entry	Cluster Pair	Chain 1	Res1#	Res1 Name	Chain 2	Res2#	Res2 Name	Distance (Å)
1	1-2	O	29	GLY	D	15	GLU	3.85
2	1-2	O	29	GLY	D	16	GLY	3.82
3	1-2	O	31	GLY	D	17	GLN	4.30
4	1-2	R	14	LEU	o	10	VAL	4.37
5	1-2	R	16	GLY	o	9	ASP	3.99
6	1-2	f	20	D6M	o	10	VAL	4.40
7	1-2	f	27	VAL	D	2	ALA	4.25
8	1-2	f	28	ARG	D	2	ALA	4.10
9	1-2	f	31	GLY	D	2	ALA	4.22
10	1-2	q	1	HIS	D	18	ALA	4.10
11	1-2	q	9	ASP	D	18	ALA	4.25
12	2-3	B	3	GLU	e	4	GLY	4.27
13	2-3	B	9	ASP	r	7	THR	4.18
14	2-3	B	11	SER	r	7	THR	4.39
15	2-3	B	11	SER	r	8	SER	4.02
16	2-3	B	12	SER	r	7	THR	4.48
17	2-3	B	19	ALA	r	23	ILE	4.27

18	2-3	B	25	TRP	r	24	ALA	3.84
19	2-3	B	31	GLY	r	16	GLY	3.86
20	2-3	T	24	ALA	r	1	HIS	3.49
21	2-3	Z	1	HIS	e	3	GLU	4.47
22	2-3	Z	3	GLU	e	2	ALA	3.71
23	2-3	g	1	HIS	e	9	ASP	4.38
24	2-3	g	18	ALA	G	19	ALA	4.44
25	2-3	g	25	TRP	r	27	VAL	4.38
26	2-3	g	26	LEU	r	28	ARG	4.11
27	2-3	g	27	VAL	r	29	GLY	4.43
28	3-4	E	5	THR	c	6	PHE	4.49
29	3-4	E	6	PHE	U	5	THR	4.32
30	3-4	E	7	THR	U	2	ALA	4.11
31	3-4	E	9	ASP	U	2	ALA	4.34
32	3-4	E	9	ASP	U	7	THR	3.93
33	3-4	E	15	GLU	U	1	HIS	3.83
34	3-4	E	23	ILE	I	31	GLY	4.45
35	3-4	G	5	THR	U	31	GLY	4.45
36	3-4	G	12	SER	i	2	ALA	3.88
37	3-4	N	1	HIS	c	11	SER	4.35
38	3-4	N	5	THR	U	4	GLY	3.69
39	3-4	P	10	VAL	i	18	ALA	4.12
40	3-4	W	1	HIS	I	18	ALA	3.93
41	3-4	W	2	ALA	c	5	THR	4.28
42	3-4	W	15	GLU	I	24	ALA	4.18
43	3-4	W	17	GLN	c	5	THR	4.44
44	3-4	W	18	ALA	I	25	TRP	4.48
45	3-4	d	17	GLN	m	19	ALA	4.01
46	3-4	d	17	GLN	m	21	GLU	4.41
47	3-4	d	26	LEU	I	28	ARG	4.50
48	3-4	d	26	LEU	I	31	GLY	4.32
49	3-4	d	27	VAL	I	28	ARG	4.23
50	3-4	d	30	ARG	I	11	SER	4.43
51	3-4	d	31	GLY	I	14	LEU	4.48
52	3-4	r	14	LEU	i	18	ALA	4.19
53	3-4	r	18	ALA	i	8	SER	3.88
54	3-4	r	19	ALA	i	11	SER	4.41
55	3-4	r	19	ALA	i	12	SER	3.89

(f) 45-mer, 300K, replicate 2

Entry	Cluster Pair	Chain 1	Res1#	Res1 Name	Chain 2	Res2#	Res2 Name	Distance (Å)
1	1-2	F	3	GLU	q	7	THR	4.37
2	1-2	F	9	ASP	q	4	GLY	4.45
3	1-2	F	10	VAL	f	8	SER	3.93
4	1-2	F	11	SER	f	9	ASP	4.15
5	1-2	F	11	SER	q	4	GLY	4.40
6	1-2	F	21	GLU	q	1	HIS	4.48
7	1-2	I	24	ALA	M	11	SER	3.78
8	1-2	g	1	HIS	f	4	GLY	4.15
9	1-2	g	10	VAL	M	20	D6M	4.18
10	1-2	j	2	ALA	q	11	SER	4.07
11	1-2	j	21	GLU	M	4	GLY	3.85
12	1-2	j	29	GLY	q	8	SER	4.07
13	2-3	G	3	GLU	e	8	SER	4.44
14	2-3	G	4	GLY	e	8	SER	3.37
15	2-3	G	4	GLY	e	9	ASP	4.11
16	2-3	G	24	ALA	e	1	HIS	4.07
17	2-3	G	31	GLY	c	19	ALA	4.25
18	2-3	K	4	GLY	L	4	GLY	4.49
19	2-3	K	4	GLY	L	12	SER	3.90
20	2-3	l	3	GLU	T	1	HIS	4.04
21	2-3	l	4	GLY	T	2	ALA	4.38
22	2-4	K	22	PHE	X	10	VAL	3.98
23	2-4	d	2	ALA	X	2	ALA	4.27
24	2-4	d	5	THR	X	29	GLY	3.99
25	2-4	d	10	VAL	X	6	PHE	4.20
26	2-4	l	2	ALA	X	4	GLY	3.94
27	2-4	l	3	GLU	X	2	ALA	3.88
28	2-4	l	20	D6M	X	10	VAL	3.37
29	3-4	c	5	THR	A	30	ARG	3.93

(g) 45-mer, 360K, replicate 1

Entry	Cluster Pair	Chain 1	Res1#	Res1 Name	Chain 2	Res2#	Res2 Name	Distance (Å)
1	1-2	F	31	GLY	f	4	GLY	4.36
2	1-2	O	6	PHE	a	31	GLY	4.41
3	1-2	Z	2	ALA	P	5	THR	4.12
4	1-2	Z	3	GLU	P	2	ALA	3.95
5	1-2	Z	12	SER	V	24	ALA	4.30
6	1-2	Z	13	TYR	V	23	ILE	4.49

7	1-2	Z	15	GLU	V	24	ALA	4.25
8	1-2	Z	19	ALA	M	8	SER	4.16
9	1-2	e	5	THR	M	19	ALA	4.45
10	1-2	i	17	GLN	P	2	ALA	4.40
11	1-3	A	1	HIS	c	15	GLU	4.42
12	1-3	A	1	HIS	c	18	ALA	3.64
13	1-3	A	17	GLN	c	17	GLN	4.10
14	1-3	A	17	GLN	r	4	GLY	4.26
15	1-3	A	18	ALA	r	7	THR	4.34
16	1-3	A	18	ALA	r	8	SER	3.61
17	1-3	A	19	ALA	r	4	GLY	4.39
18	1-3	A	31	GLY	c	4	GLY	4.26
19	1-3	B	9	ASP	c	4	GLY	4.22
20	1-3	B	11	SER	c	5	THR	3.98
21	1-3	B	24	ALA	c	6	PHE	4.31
22	1-3	B	27	VAL	L	31	GLY	4.27
23	1-3	B	29	GLY	L	25	TRP	3.36
24	1-3	O	7	THR	p	5	THR	4.42
25	1-3	O	8	SER	p	9	ASP	4.43
26	1-3	O	11	SER	H	5	THR	4.27
27	1-3	O	22	PHE	H	4	GLY	4.46
28	1-3	O	22	PHE	H	24	ALA	3.90
29	1-3	O	29	GLY	c	9	ASP	3.84
30	1-3	U	4	GLY	L	3	GLU	3.56
31	1-3	U	4	GLY	p	19	ALA	4.36
32	1-3	U	5	THR	p	19	ALA	4.20
33	1-3	U	10	VAL	r	7	THR	4.06
34	1-3	Z	31	GLY	N	4	GLY	4.35
35	2-3	M	2	ALA	m	1	HIS	4.36
36	2-3	M	2	ALA	m	2	ALA	4.47
37	2-3	M	4	GLY	m	2	ALA	3.49
38	2-3	M	7	THR	E	15	GLU	3.98
39	2-3	M	7	THR	E	16	GLY	4.28
40	2-3	V	24	ALA	K	29	GLY	4.34
41	2-3	a	2	ALA	K	3	GLU	4.35
42	2-3	a	3	GLU	m	18	ALA	4.14
43	2-3	a	4	GLY	K	4	GLY	3.58
44	2-3	a	4	GLY	m	16	GLY	4.50
45	2-3	a	7	THR	m	25	TRP	4.11
46	2-3	a	10	VAL	p	29	GLY	3.71

47	2-3	a	18	ALA	K	7	THR	3.80
48	2-3	a	18	ALA	K	15	GLU	4.36
49	2-3	a	19	ALA	K	15	GLU	3.81
50	2-3	h	17	GLN	E	30	ARG	4.10
51	2-3	h	22	PHE	R	24	ALA	3.75
52	2-3	h	24	ALA	E	19	ALA	4.00
53	2-3	h	24	ALA	E	21	GLU	3.98
54	2-3	o	3	GLU	R	2	ALA	3.57
55	2-3	s	8	SER	K	15	GLU	4.33

(h) 45-mer, 360K, replicate 2

Entry	Cluster Pair	Chain 1	Res1#	Res1 Name	Chain 2	Res2#	Res2 Name	Distance (Å)
1	1-2	F	3	GLU	e	2	ALA	4.02
2	1-2	F	4	GLY	e	3	GLU	3.79
3	1-2	P	2	ALA	N	2	ALA	3.74
4	1-2	P	5	THR	J	4	GLY	4.05
5	1-2	P	5	THR	J	8	SER	3.68
6	1-2	P	7	THR	J	4	GLY	4.37
7	1-2	P	29	GLY	J	2	ALA	4.14
8	1-2	P	30	ARG	p	29	GLY	4.14
9	1-2	V	10	VAL	e	9	ASP	4.39
10	1-2	h	8	SER	l	29	GLY	4.31
11	1-2	h	9	ASP	l	4	GLY	4.14
12	1-2	h	17	GLN	Y	2	ALA	4.50
13	1-2	h	19	ALA	e	10	VAL	4.29
14	1-2	h	21	GLU	e	12	SER	4.18
15	1-2	h	25	TRP	J	6	PHE	4.41
16	1-2	h	29	GLY	N	9	ASP	3.95
17	1-2	h	31	GLY	N	1	HIS	3.89
18	1-2	j	1	HIS	l	7	THR	4.44
19	1-3	C	1	HIS	i	3	GLU	3.71
20	1-3	C	3	GLU	A	17	GLN	4.31
21	1-3	C	5	THR	A	18	ALA	4.17
22	1-3	C	6	PHE	A	19	ALA	3.49
23	1-3	M	17	GLN	H	9	ASP	4.41
24	1-3	Q	1	HIS	W	12	SER	4.18
25	1-3	S	2	ALA	a	9	ASP	4.27
26	1-3	S	3	GLU	H	2	ALA	3.97
27	1-3	S	4	GLY	H	2	ALA	3.58
28	1-3	S	5	THR	i	19	ALA	4.18

29	1-3	S	21	GLU	H	1	HIS	4.44
30	1-3	X	4	GLY	W	24	ALA	4.49
31	1-3	X	4	GLY	W	28	ARG	4.24
32	1-3	X	6	PHE	W	20	D6M	4.01
33	1-3	X	11	SER	i	30	ARG	4.43
34	1-3	X	18	ALA	i	29	GLY	3.67
35	1-3	X	30	ARG	i	2	ALA	4.13
36	1-3	c	19	ALA	H	3	GLU	4.07
37	1-3	c	19	ALA	H	5	THR	3.84
38	1-3	o	3	GLU	W	8	SER	3.92
39	1-3	o	5	THR	W	6	PHE	4.08
40	1-3	r	5	THR	i	16	GLY	4.28
41	1-4	F	1	HIS	G	16	GLY	4.37
42	1-4	F	2	ALA	G	17	GLN	3.95
43	1-4	I	2	ALA	B	19	ALA	4.46
44	1-4	I	19	ALA	O	21	GLU	4.13
45	1-4	L	2	ALA	B	4	GLY	4.39
46	1-4	L	2	ALA	G	4	GLY	4.40
47	1-4	L	5	THR	B	2	ALA	4.41
48	1-4	L	5	THR	B	24	ALA	4.45
49	1-4	L	6	PHE	B	24	ALA	3.59
50	1-4	L	8	SER	G	7	THR	4.48
51	1-4	L	9	ASP	G	8	SER	3.93
52	1-4	S	15	GLU	B	29	GLY	4.03
53	1-4	S	24	ALA	B	29	GLY	3.06
54	1-4	S	30	ARG	B	27	VAL	4.46
55	1-4	S	31	GLY	B	1	HIS	4.46
56	1-4	j	2	ALA	U	9	ASP	4.00
57	1-4	j	2	ALA	U	29	GLY	4.40
58	1-4	j	28	ARG	O	16	GLY	4.35
59	2-4	K	16	GLY	q	7	THR	3.76
60	2-4	K	21	GLU	q	4	GLY	4.29
61	2-4	K	25	TRP	D	11	SER	4.41
62	2-4	K	26	LEU	D	10	VAL	4.36
63	2-4	Y	8	SER	G	17	GLN	4.05
64	2-4	n	15	GLU	U	11	SER	4.46

S7. Enhanced Sampling to Evaluate the Convergence of Simulation Process and Stability of Conformers

The radius of gyration, a metric that quantifies the spatial distribution of atoms, was used to assess oligomerization progression over time (**Fig. S6**). A large initial Rg indicates monomers randomly distributed within the simulation box, while its subsequent decrease signified oligomerization. A plateau at the end of the Rg curve suggests structural convergence.

Simulation strucutre can be trapped in a local minima, so the observed state may not represent an equilibrated or a global minimum energy state. To overcome this issue, we employed an enhanced sampling method, simulated annealing (SA), to evaluate whether the simulations had reached structural equilibrium over iterative annealing cycles.[1] Each SA simulation involved 10 annealing cycles, with each cycle comprising 700 ns of cooling and 300 ns of heating. A 10 ns interval was applied between cycles to allow gradual temperature transitions.

Both the SA and constant temperature simulations yielded similar results; the Rg converged by the end of each simulation, demonstrating that the system reached equilibrium. **Table S5** details the resulting SA structures, which closely resembled those from constant temperature simulations, exhibiting well-defined hydrophobic cores and forming two to four subunit clusters.

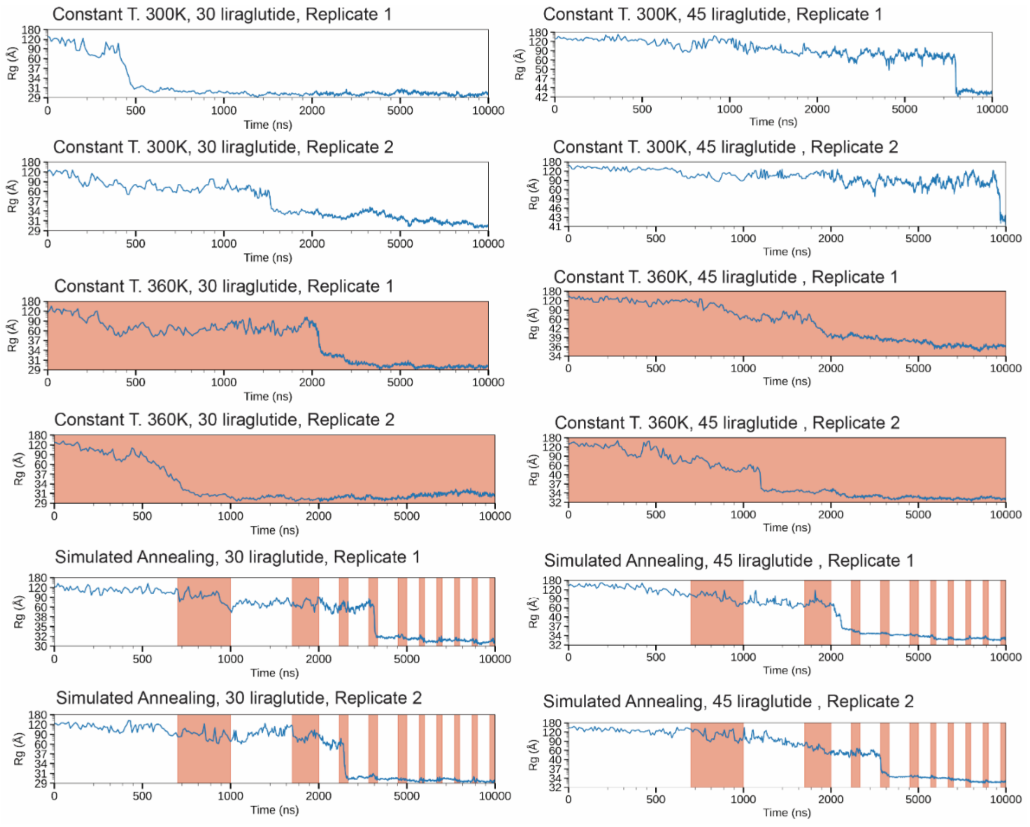

Figure S6. Molecular dynamics simulation (MDS) trajectory for 30 or 45 liraglutide units under simulated annealing (SA) or constant temperature conditions. Orange bands indicate heating intervals where the system temperature was raised to 360 K.

Table S5. Summary of end-point structures from four simulated annealing (SA) results.

Condition	Structure	Hydrophobic Contacts Contribution (%)	Hydrophilic Contacts Contribution (%)	Hybrid Contacts Contribution (%)	Subunit Composition (w-x-y-z)	Averaged Subunit Size (n)
30mer-Simulated Annealing-Rep1	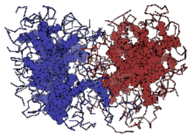	11	56	33	14-16	15 ± 1
30mer- Simulated Annealing -Rep2	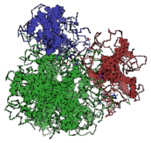	19	47	34	5-6-19	10 ± 8
45mer- Simulated Annealing -Rep1	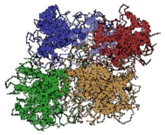	17	37	46	7-11-12-15	11 ± 3
45mer- Simulated Annealing -Rep2	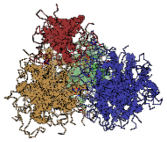	8	52	40	6-10-14-15	11 ± 4

REFERENCE

(1) Bernardi, R. C.; Melo, M. C. R.; Schulten, K. Enhanced sampling techniques in molecular dynamics simulations of biological systems. *Biochim Biophys Acta* **2015**, *1850* (5), 872-877. DOI: 10.1016/j.bbagen.2014.10.019 From NLM Medline.
